# Population-based Prostate Cancer Screening in Kazakhstan

**Published:** 2017-07

**Authors:** Yevgeniy ISHKININ, Alma ZHYLKAIDAROVA, Nurzhan NURGALIYEV, Elmira AUYEZOVA, Ainash OSHIBAYEVA, Nadezhda GORBUNOVA

**Affiliations:** 1. Kazakh Institute of Oncology and Radiology, Almaty, Kazakhstan; 2. Medical University “High School of Public Health”, Almaty, Kazakhstan; 3. Al-Farabi Kazakh National University, Almaty, Kazakhstan

**Keywords:** Prostate cancer, PSA, PHI, Population-based screening, Early diagnosis

## Abstract

**Background:**

Issues of mass screening for prostate cancer rather controversial since 2013 in 11 regions of Kazakhstan introduced a population-based screening for prostate cancer, so we need to evaluate its results.

**Methods:**

In different regions of Kazakhstan during 2013–2015, a total of 321548 prostate-specific antigens (PSA) were determined in men aged 50–66 yr, under the Prostate Health Index (PHI) and transrectal ultrasonography (TRUS) guided prostate biopsy with histological examination.

**Results:**

PSA level up to 4 ng/ml in 310870 (96.7%) men, PSA level between 4 and 10 ng/ml in 8 624 (2.7%) men, PSA level above 10 ng/ml in 2054 (0.6%) men. PHI was identified in 5716 (1.8%) men, of which 2867 cases were with PHI ≥ 25 (35.9%). Totally, 3680 biopsies (1.1%) of the prostate were performed. As part of the screening, 2870 cases (0.88%) of benign prostatic hyperplasia and prostatic intraepithelial neoplasia were found. Of 742 cases of prostate cancer (0.23%) were revealed. The stages of prostate cancer screening were as follows: stage I in 172 men (23.2%), stage II in 444 men (59.8%), stage III in 98 men (13.2%) and stage IV in 28 (3.8%) men. The indicators of prostate cancer early diagnosis in the I–II stages were bigger in the “screening regions” than in the “traditional diagnostics” regions: RR 1.35 95% CI (1.24 – 1.46), OR 1.84 95% CI (1.58–2.15). Prostate cancer was detected at I–II stages in the “screening” regions only by screening vs traditional diagnostics, with RR 1.64 95% CI (1.56 – 1.73), OR 4.77 95% CI (3.87–5.87).

**Conclusion:**

Implementation of screening can improve the diagnosis of prostate cancer in the early stages.

## Introduction

According to the GLOBOCAN database (1), 1.1 million males in 2012 were diagnosed with prostate cancer; which is in the second place in the structure of oncologic diseases (13.6%) in the world with mortality rate of 307 thousand patients (6.6%). An average world morbidity was 31.1 per 100000 people with a mortality rate of 7.8 per 100000 people. An average ratio of mortality to morbidity around the world is 25.1%. Prostate cancer morbidity tends to increase over the last years in Kazakhstan as well. Thus, in 2001, 534 males (3.6 per 100000 men) were diagnosed with prostate cancer, while in 2012 their number amounted to 597 (5.3 per 100000 men). High mortality rate among patients with prostate cancer is dynamically growing, but the growth rate has been stable: in 2001, 296 patients died (2.0 per 100000 men) while in 2012, 394 people passed away (2.3 per 100000 men). In 2012, the ratio of mortality to morbidity in Kazakhstan was 43.4%, which is due to the late diagnostics of prostate cancer. The trend, however, has been improving over the last years: in 2001, there were 76.1% patients with a newly diagnosed prostate cancer in the third and fourth stages, whereas in 2012 this number decreased to 57.2% of patients. This leads to the lowest 5-year survival rate among all oncologic pathologies in Kazakhstan in 2012-only 29.2% of patients with prostate cancer live longer than five years (2).

Following the Health Development State Program of the Republic of Kazakhstan, prostate cancer screening program was launched in 2013, given the trend towards low ratio of mortality to morbidity and low 5-year survival rate due to late diagnosis. According to WHO Regional Office for Europe, introduction of mass screening programs at a national level remains rather controversial (3), so the initial results of population-based screening for prostate cancer need to be assessed.

## Materials and Methods

Since 2013, 11 of the 16 regions of Kazakhstan introduced the screening for prostate cancer in males aged 50–66 yr, with a 4 year interval. Patients are informed about the advantages and disadvantages of screening in the course of a wide information campaign, in compliance with the principle of voluntarism. It is obligatory to inform the patients about the requirements for quality blood samples. A method of screening is an immuno chemiluminometric assay (ICMA) for PSA. Primary health care staff takes the blood samples for the pre-analytical processing and delivery to the central laboratory in each region equipped with ICMA analyzers. The methods for in-depth diagnosis are as follows: the PHI is identified when the value of total PSA is at the level of 4–10 ng/ml by Hybritech calibration. The method of TRUS with 8 cores prostate biopsy is used if the patient’s PHI is ≥ 25, and in the patients with PSA ≥ 10 ng/ml. Histomorphology of biopsy material is examined in the pathology lab of an oncologic center or a central regional pathology office following the generally accepted international standards and classification with indication of the Gleason score (4).

In 2013, the prostate cancer screening was carried out in East Kazakhstan, West Kazakhstan, Kyzylorda and Pavlodar regions and the cities of Almaty and Astana. In 2014, this work was continued in the Aktobe, Atyrau, Karaganda, Kostanai and North Kazakhstan regions. In 2015, the screening continued in all the above mentioned regions. The rest five regions of Kazakhstan follow “the traditional diagnosis of prostate cancer” (a standard workout by request). The data of Kazakhstan Cancer Registry from 2001 to 2015 and the MedInform Company reports on the results of preventive medical examinations for prostate cancer according to the forms completed in the primary care organizations were used for the analysis. The dynamics of prostate cancer was analyzed using Kazakhstan Cancer Registry, official reports of the regional oncologic centers on primary patients registered in 2013–2015 and notices about patients with first-ever diagnosis of prostate cancer for the 2013–2015.

## Results

PSA level was examined in 321548 men during 2013–2015, as part of a screening study. Total PSA level up to 4 ng/ml was identified in 310870 males (96.7%), the level of total PSA between 4 to 10 ng/ml - in 8624 men (2.7%), and total PSA level above 10 ng/ml was found in 2054 men (0.6%). At the same time, hemolysis or chylesis observed in the blood samples of 1604 men (0.5%) were due to non-compliance with pre-analytical phase of the algorithm. The patients had to reproduce blood samples for PSA. PHI is found in only 5716 men (1.8% of those surveyed), which comprised only 66.3% of the 8624 men in whom PHI had to be identified according to the screening algorithm, of these 2867 cases (35.9%) had a level of PHI ≥ 25. Total number of prostate biopsies was 3680 (1.1%), making 74.8% of the indicated, according to the screening algorithm (for 2054 males with total PSA levels higher than 10 ng/ml and 2867 patients with PHI level≥25). The screening revealed 2870 cases (0.88%) of benign prostatic hyperplasia and prostatic intraepithelial neoplasia, it revealed also 742 cases of prostate cancer (0.23% of those surveyed). To reveal one patient with prostate cancer 433.3 total PSA tests 7.7 PHI level measurements and 5 biopsies with histological examination were conducted.

**Fig. 1:**
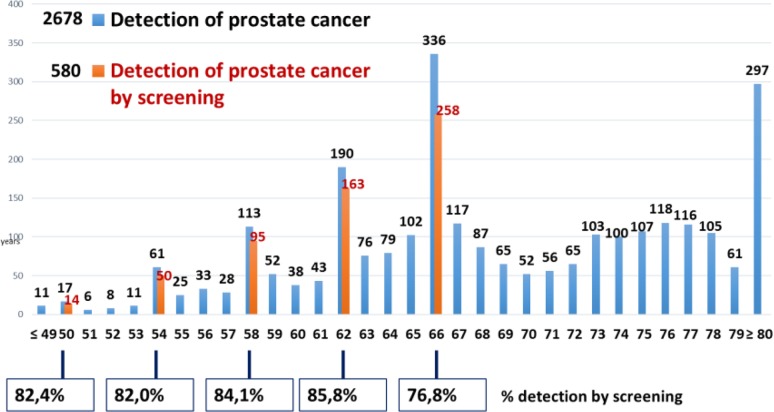
Distribution by ages of newly diagnosis prostate cancer in 2014–2015 yr

**Fig. 2:**
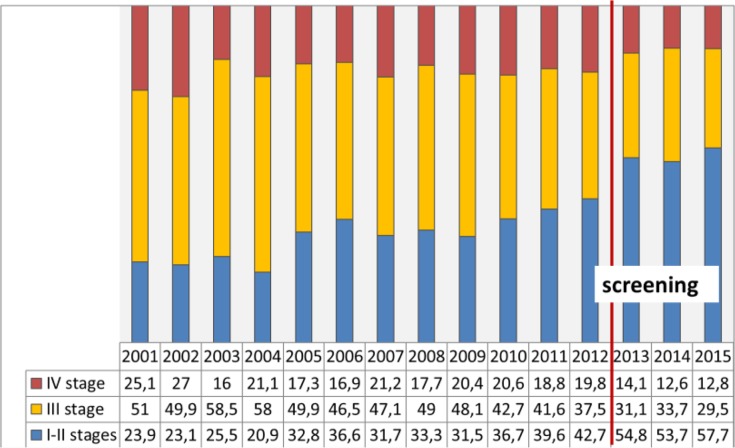
Distribution of newly diagnosis prostate cancer percentage by stages in 2001–2015 years, Cancer Registry data

**Fig. 3:**
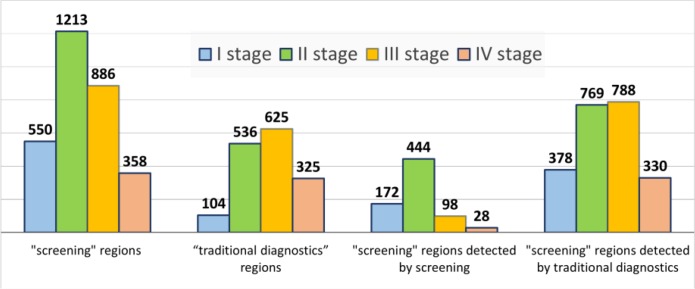
Distribution of newly diagnosed prostate cancer by stages according to screening conducted in 2013–2015

After the introduction of screening for 3 yr the epidemiological situation in Kazakhstan has changed, with an increased morbidity of 2.5 per 100000 people per year, or 480 newly diagnosed patients, whereas the ratio of mortality to morbidity increased by 15.2% and amounted to 28.2%. The ratio of mortality to morbidity in the regions of “screening” was 25.5%, while in the regions with traditional diagnostics it made 46.7%. An offset age-peak detection of prostate cancer showed a shift from 70–74 yr in 2012 to the screening age 62–66 yr in 2013, 2014 and 2015. In this relatively young age there is a lower incidence of co-morbidities and a high probability of radical treatment of prostate cancer patients. In the regions with traditional diagnostics, there is no change by age in the cohort of patients. In 2014 and 2015 yr (Fig. 1), analysis of the prostate cancer detection by age marked the age peaks for the screening target group, with a large percentage of detection of prostate cancer by screening. Meanwhile, the early diagnosis of prostate cancer improved at stages I–II by 14.9% (from 42.8% in 2012 to 57.7% in 2015) (Fig. 2). In 2013–2015, the indicators of early diagnosis of prostate cancer in the regions where screening was performed were higher against those of the regions with “traditional diagnosis”. There is a higher level of the prostate cancer detection in the regions under the screening programme than in the regions with “traditional diagnosis” (Fig. 3).

In the 11 regions where the screening programme was carried out, the prostate cancer was found in 1763 males (58.6%) in the I–II stages, while 1244 (41.4%) screened patients revealed the III–IV stages of the prostate cancer. In the 5 regions where the screening was not carried out, the prostate cancer was detected in 372 men (43.5%) at the I–II stages, with 483 prostate cancer cases at the stages III–IV (56.5%). RR 1.35 95% CI (1.24 – 1.46), OR 1.84, 95% CI (1.58–2.15) Total 6.16 cases should be screened in order to prevent detection of one prostate cancer case in the III–IV stages among all men. The screening allowed to reveal 616 (83%) cases of prostate cancer at stages I–II and 126 (17.0%) at stages III–IV. In these same regions, methods of “traditional diagnosis” revealed 1147 (50.7%) at stages 1118, and I–II (49.3%) at stages III–IV. RR 1.64 95% CI (1.56 – 1.73), OR 4.77 95% CI (3.87–5.87). Total 3.09 cases should be screened in order to prevent detection of one prostate cancer case in the III–IV stages in the men of 50, 54, 58, 62 and 66 yr of age.

## Discussion

Determining the level of PSA has revolutionized the diagnosis of prostate cancer (5). Only two large multicenter randomized trials to assess the effect of screening on mortality from prostate cancer were conducted in United States of America (USA) and Europe: “Prostate, Lung, Colorectal and Ovarian (PLCO) Cancer Screening Trial” and “European Randomized Study of Screening for Prostate Cancer (ERSPC)”. The PLCO study was conducted in 10 states and included 76693 patients aged 55–74 (6). The screening was carried out using a blood test for PSA (in the 4 states) and digital rectal examination (in the 6 states). No significant statistical differences and data on the effectiveness of the screening for prostate cancer have been received (7). The ERSPC study included 162243 men aged 55–69; after 9 yr of monitoring the mortality rate from prostate cancer in the screening group was lower by 20%, while after 10-year follow-up it was 30% lower than that of the control group (8). However, there is a high risk of over diagnosis, and many researchers have questioned the need for large-scale population-based screening due to its high expenses (9–13). Anyway, the situation with the early diagnosis of prostate cancer in Kazakhstan is not comparable with the diagnosis in the USA and Europe. Therefore, the screening of prostate cancer in the Republic of Kazakhstan is more efficient than PLCO, ERSPC. It would serve as a model for other countries with similar epidemiological problem of advanced prostate cancer. Patients’ diagnosed prostate cancer through screening saves their lives (14). Men want to participate actively in decisions affecting their health, to be fully informed on all issues, including the identified levels of PSA (15). Men believe that dissemination of this information is important (16).

In recent years, the problem of defining specific forms of PSA is of great interest in screening programs. One of these forms is known as a pro-enzyme form of PSA (pro PSA). Pro PSA, and in particular its truncated form -2pro PSA may be more associated with prostate cancer, compared to the PSA and the use of these forms and relations to the PSA can improve the detection of prostate cancer (17). Compared with free PSA parameter, the -2proPSA parameter, expressed as a ratio to the free forms of PSA PHI, is more effective in detecting prostate cancer in cases where the PSA is between 4 to 10 ng/ml. In this case, the value of PHI ≥25 implies a higher probability of prostate cancer or precancerous conditions. According to the algorithm of population screening for prostate cancer in Kazakhstan at the level of total PSA of 4–10 ng/ml, and PHI ≥ 25, a needle biopsy of the prostate should be carried out, whereas at the level of PHI <25 a dynamic control should be maintained.

This is the first screening in Kazakhstan, with the target group of only men. Its implementation contributes to men’s responsibility for their health, cancer awareness and consolidating efforts to protect men’s health.

## Conclusion

The introduction of population screening for prostate cancer in Kazakhstan has improved the diagnosis of prostate cancer in the early stages.

## Ethical considerations

Ethical issues (including plagiarism, informed consent, misconduct, data fabrication and/or falsification, double publication and/or submission, redundancy, etc.) have been completely observed by the authors
